# Variability in Treatment for Carbon Monoxide Poisoning in Japan: A Multicenter Retrospective Survey

**DOI:** 10.1155/2018/2159147

**Published:** 2018-12-04

**Authors:** Motoki Fujita, Yasutaka Oda, Kotaro Kaneda, Yoshikatsu Kawamura, Takashi Nakahara, Masaki Todani, Takeshi Yagi, Yasutaka Koga, Ryosuke Tsuruta

**Affiliations:** ^1^Acute and General Medicine, Yamaguchi University Graduate School of Medicine, 1-1-1 Minamikogushi, Ube, Yamaguchi 755-8505, Japan; ^2^Advanced Medical Emergency and Critical Care Center, Yamaguchi University Hospital, 1-1-1 Minamikogushi, Ube, Yamaguchi 755-8505, Japan

## Abstract

**Background:**

The aim of this study was to identify practice differences in the treatment of carbon monoxide (CO) poisoning with or without hyperbaric oxygen (HBO_2_) therapy in Japan.

**Materials and Methods:**

Using an online survey website (Google form), we created a questionnaire and invited interested institutions to join the COP-J Study, a prospective observational study of CO poisoning in Japan.

**Results:**

Forty-eight (63%) of 76 institutions replied to the questionnaire. Thirty-three institutions (69%) administered HBO_2_ therapy to patients with CO poisoning, and 15 institutions (31%) did not. Consciousness disturbance on arrival, exposure to CO for a long time, and elevation of arterial carboxyhemoglobin (CO-Hb) were the major indications for HBO_2_ therapy. The maximum therapeutic pressures were 2.0, 2.5, and 2.8 atmospheres absolute (ATA) at 19 (58%), 6 (18%), and 8 (24%) institutions, respectively. The number of HBO_2_ sessions on the first day was 1–3, and 1–7 sessions were administered on days 2–7. Seventeen (35%) institutions treated patients with delayed neurological sequelae (DNS) and 15 of them used HBO_2_ therapy for DNS.

**Conclusions:**

This survey indicates that HBO_2_ therapy for CO poisoning was varied in both the indications and practice regimens used in Japan.

## 1. Introduction

Based on the results of a randomized controlled trial (RCT) reported by Weaver et al. [1], hyperbaric oxygen (HBO_2_) therapy is thought to be essential to prevent delayed neurological sequelae (DNS) in patients with carbon monoxide (CO) poisoning. In the United States and Europe, the clinical use of HBO_2_ therapy for CO poisoning is reported to vary, despite several guidelines published by scientific institutions, such as the Undersea and Hyperbaric Medical Society (UHMS) and the European Committee for Hyperbaric Medicine (ECHM) [2, 3]. It is unclear how HBO_2_ therapy is used for CO poisoning in Japan.

We recently began a prospective observational study of CO poisoning in Japan, the “COP-J Study,” to clarify the effects of HBO_2_ therapy in the acute phase of CO poisoning. The COP-J Study was approved by the Japanese Society of Intensive Care Medicine (JSICM) (no. 0011). Members of the JSICM, the Japanese Society for Clinical Toxicology, the Japanese Society of Hyperbaric and Undersea Medicine, and/or the Japanese Association for Acute Medicine were invited to participate. We called for their participation at the 31st Japanese Association for Acute Medicine Chugoku-Shikoku district meeting, the 6th Japanese Undersea and Hyperbaric Medical Society Chugoku-Shikoku district meeting, the 50th annual meeting of Japanese Undersea and Hyperbaric Medical Society, and the 37th annual meeting of Japanese Society for Clinical Toxicology. A letter of invitation to the COP-J Study was sent to all 288 certified training institutions of the JSICM and to all councilors of the Japanese Undersea and Hyperbaric Medical Society Chugoku-Shikoku district meeting. This survey was performed before the COP-J Study was commenced, to identify practice differences in the treatment of CO poisoning with HBO_2_ therapy in Japan.

## 2. Methods

This study was conducted between March 2016 and July 2016. We created a questionnaire with an online survey website (Google form). An e-mail containing a link directing the responders to the survey website was sent to a responsible person at the institutions that agreed to participate in the COP-J Study. The questionnaire consisted of the following questions: data recorded in the most recent year were collated.

1. What type of HBO_2_ chamber do you have?
2. How many patients do you treat for CO poisoning a year?
3. Do you use HBO_2_ therapy for CO poisoning?
4. Which criteria do you consider to be indications for HBO_2_ therapy in patients with CO poisoning?
5. How much pressure do you use in HBO_2_ therapy for CO poisoning?
6. How many rounds of HBO_2_ therapy do you administer to CO poisoning patients in the 24 h after admission?
7. How many rounds of HBO_2_ therapy do you administer to CO poisoning patients between day 2 and day 7 after admission?
8. How long do you administer oxygen to CO poisoning patients?
9. How long does a patient with CO poisoning remain in bed?
10. Is magnetic resonance imaging (MRI) of the brain performed in the acute phase?
11. If yes, is the treatment altered based on the MRI findings?
12. If yes, what parts of the treatment are altered based on the MRI findings?
13. Do you treat patients with delayed neurological sequelae (DNS)?
14. If yes, do you use HBO_2_ therapy for DNS?

The results were assessed with basic descriptive analyses. We calculated the percentages of the responses to each question, using the number of centers that responded to that particular question as the denominator. Percentages were rounded to the nearest integer value.

## 3. Results

A total of 76 institutions received the invitation to participate in this survey and 48 (63%) institutions responded. Of these 48 institutions, 28 (58%) were university hospitals, 13 (27%) were general hospitals with emergency and critical care centers, and 7 (16%) were general hospitals without emergency or critical care centers. Twenty-one (44%) and 10 (21%) institutions had monoplace and multiplace HBO_2_ chambers, respectively (Table 1). Seventeen (35%) institutions had no HBO_2_ chamber (Table 1). The numbers of patients with CO poisoning per year were 0–1 patient at 12 institutions (12%), 2–5 patients at 18 institutions (37%), 6–9 patients at 10 institutions (21%), and >10 patients at 8 institutions (17%; Table 2).

Thirty-three institutions (69%), including two institutions without an HBO_2_ chamber, administered HBO_2_ therapy to patients with CO poisoning, whereas 15 institutions (31%) did not. The two institutions that did not have an HBO_2_ chamber administered HBO_2_ therapy by transferring the patients to another hospital that had an HBO_2_ chamber. The indications for HBO_2_ therapy in CO poisoning patients at the 33 institutions are shown in Table 3. Consciousness disturbance upon arrival, exposure to CO for a long time (>12 hours), and elevation of arterial carboxyhemoglobin (CO-Hb) were the major indications for HBO_2_ therapy. Others included myocardial injury, age >60 years, organ failure, and nonmechanical ventilation.

The maximum therapeutic pressure used for HBO_2_ therapy and the number of HBO_2_ sessions during the first 24 h and on days 2–7 varied across institutions (Table 4). The maximum therapeutic pressures were 2.0, 2.5, and 2.8 atmospheres absolute (ATA) in 19 (58%), 6 (18%), and 8 (24%) institutions, respectively (Table 4(a)). The number of HBO_2_ sessions was 1–3 during the first 24 h (Table 4(b)) and 0–7 during the following 6 days (Table 4(c)). No institution continued HBO_2_ therapy after the first week.

Table 5 lists the durations of oxygen administration and the period of bed rest for CO poisoning patients. The majority of institutions (28 of 48, 58%) administered oxygen to CO poisoning patients until their CO-Hb levels had normalized. Several institutions used other criteria to select the period of oxygen administration, including for 24 h after CO exposure, for >24 h after CO exposure, until the next morning, and until the recovery of consciousness. All institutions had some criterion or criteria for the duration of oxygen administration. However, 22 (46%) institutions had no criterion for the duration of bed rest.

An MRI scan of the brain was performed in all CO poisoning patients at 14 (29%) institutions and was performed according to the severity of poisoning at 30 (66%) institutions. Brain MRI was not performed at four (8%) institutions. Of the 44 institutions that performed brain MRI for CO poisoning patients, 13 (30%) institutions altered the treatment if the findings were abnormal. The changes to treatment were increased length of follow-up (five institutions), addition of HBO_2_ therapy (three institutions), and administration of edaravone (two institutions).

Seventeen (35%) institutions treated patients with DNS and 15 of these performed HBO_2_ therapy for DNS.

## 4. Discussion

This survey demonstrates that, in Japan, the treatment of patients with acute CO poisoning varies, including the inclusion of HBO_2_ therapy. HBO_2_ therapy was performed for CO poisoning in 69% of the institutions surveyed, and the HBO_2_ therapy profiles used to treat patients with CO poisoning varied widely. These results suggest that there is no consensus regarding the treatment for acute CO poisoning, including the administration of HBO_2_ therapy, in Japan.

In this survey, the majority of centers (69%) gave HBO_2_therapy to patients with acute CO poisoning. Although it was reported that HBO_2_ therapy improved the neurological outcomes of patients with acute CO poisoning [1], there is no clinical consensus regarding HBO_2_ therapy for acute CO poisoning. The American College of Emergency Physicians Clinical Policies recommends that “emergency physicians should use HBO_2_ therapy or high-flow normobaric therapy for acute CO-poisoned patients” as a level B recommendation [4]. However, Hampson et al. wrote a rebuttal on HBO_2_therapy for acute CO poisoning [5]. They recommended that “HBO_2_ should at least be considered for all patients with acute, symptomatic CO poisoning.” In Japan, the same situation is present and there is no consensus on when to include HBO_2_ therapy for CO poisoning patients. The most recent Japanese survey revealed that only 42% of emergency and critical care centers, which usually treat patients suffering acute poisoning (including CO poisoning), had an HBO_2_ chamber [6]. This limited availability of HBO_2_ chambers might explain the low rate of HBO_2_ therapy for CO poisoning in Japan.

In this survey, the major indications for HBO_2_ therapy were consciousness disturbance upon arrival, exposure to CO for a long time, and elevated arterial CO-Hb, whereas at 24% of institutions, HBO_2_ therapy was administered to all patients diagnosed with CO poisoning. Compared with a European survey [3], very few institutions included criteria such as myocardial injury or pregnancy. These results might suggest that few Japanese institutions follow the ECHM consensus [7] or the UHMS committee report [8], reflecting the lack of consensus on HBO_2_ therapy for CO poisoning in Japan.

The present survey revealed many variations in the HBO_2_ therapy profiles for CO poisoning. The maximum therapeutic pressure and number of HBO_2_ sessions in the first 24 h or the first week were not consistent among the 33 (69%) institutions that performed HBO_2_ therapy for CO poisoning. These results are similar to reports from Europe and USA [3, 4]. The majority (58%) of the institutions performed HBO_2_ therapy at 2.0 ATA, although studies have demonstrated that HBO_2_ therapy at 2.0 ATA is not an effective treatment for CO poisoning [9, 10]. No Japanese institutions treat patients with HBO_2_ at 3.0 ATA. However, HBO_2_ at 3.0 ATA was used in a positiveRCT reported by Weaver et al. [1] and HBO_2_ at 2.5–3.0 ATA was recommended for patients with acute CO poisoning and neurological symptoms [11]. This finding may be related to the fact that 2/3 of the 31 institutions that had an HBO_2_ chamber had only a monoplace chamber and that, until 2012, monoplace chambers were restricted to therapeutic pressures ≤2.0 ATA by the Japanese Society of Hyperbaric and Undersea Medicine, if they were pressurized with oxygen. It seems likely that many institutions have continued using the same therapeutic pressure since 2012.

The number of HBO_2_ sessions performed within the first 24 h was 1–3, and the majority of institutions performed 1–7 sessions in the following 6 days. These results might be related to the health care fee for HBO_2_ therapy in Japan, although no effective regimen for HBO_2_ therapy for CO poisoning has achieved consensus in either Europe or the USA [2, 3]. In Japan, institutions can claim higher fees for HBO_2_ therapy only once per day within the first 7 days from the onset of CO poisoning than they can claim thereafter. Therefore, 1/3 of institutions might limit the number of HBO_2_ sessions to one on the first day and many institutions might continue HBO_2_ therapy until day 7. These results suggest that it might be important to standardize the HBO_2_ protocol for CO poisoning in Japan.

The majority (58%) of institutions continued oxygen administration until the CO-Hb level normalized, while almost half (46%) of the institutions did not specify the duration of bed rest. These results reflect the absence of a consensus on the duration of oxygen administration and the period of bed rest for patients with CO poisoning in Japan. Further studies are needed to establish consensus on these issues.

The survey revealed that brain MRI was performed in all CO poisoning patients at 14 (29%) institutions, and 30 (63%) institutions used brain MRI depending on the severity of poisoning. MRI is a very important examination for CO poisoning patients. It is reported that both diffusion-weighted imaging (DWI) and diffusion tensor imaging (DTI) in the acute phase are useful for predicting the onset and outcome of DNS after acute CO poisoning [12–15]. In addition, Japanese institutions have easy access to MRI because there are 51.7 MRI units per million population in Japan, which is the highest rate in the world [16].

Only 17 (35%) institutions treated patients with DNS in this survey. This suggests that it is difficult for emergency departments to follow up acute CO poisoning patients for long periods because there is a considerable interval before the onset of DNS. Of these 17 institutions, 15 performed HBO_2_ therapy for DNS. HBO_2_ therapy is reported to be useful in treating DNS [17], and it was recently reported that the combined application of HBO_2_ therapy and dexamethasone or* N*-butylphthalide improves the neurological outcome of DNS [18, 19]. However, there has been no multicenter randomized control trial of HBO_2_ therapy for DNS. Further research is required to clarify the effects of HBO_2_ therapy on DNS.

A limitation of this survey was that the survey was only conducted in those institutions that responded to the invitation to join the COP-J Study. These institutions might not be representative of institutions that treat patients with CO poisoning in Japan.

## 5. Conclusion

Our results show that, in Japan, HBO_2_ therapy for CO poisoning patients varies in not only the indications for treatment but also the practice regimens used. These results are similar to the trends reported in Europe and USA. It might be necessary to standardize the protocol for treating CO poisoning, including the indication and regimen of HBO_2_ therapy, in Japan.

## Figures and Tables

**Table 1:** Type of HBO_2_ chamber.

	Number	%
Monoplace	21	44
Multiplace	10	21
None	17	35

**Table 2:** Number of patients with CO poisoning per year.

Number of patients	Number	%
0 –1	12	25
2–5	18	37
6–9	10	21
> 10	8	17

**Table 3:** Indications for HBO_2_ therapy in patients with CO poisoning at 33 institutions.

	Number	%
Consciousness disturbance on arrival	19	58
Exposure to CO for > 12 hours	17	52
CO-Hb ≥ 10%	11	33
CO-Hb ≥ 20%	9	27
All cases	8	24
Other	5	15

Multiple answers allowed.

**Table:** (a) Maximum therapeutic pressure for HBO_2_ therapy at 33 institutions

	Number	%
2.0 ATA	19	58
2.5 ATA	6	18
2.8 ATA	8	24

**Table:** (b) Number of HBO_2_ therapy sessions during 24 h after admission at 33 institutions

	Number	%
1	12	36
2	11	33
3	8	24
Other	2	6

**Table:** (c) Number of HBO_2_ therapy sessions from day 2 to day 7 after admission at 33 institutions

	Number	%
None	6	18
1–3	13	39
4–7	11	33
Other	3	9

**Table 5:** Duration of oxygen administration and period of bed rest for CO poisoning patients.

	Duration of oxygen administration		Duration of bed rest	
	Number	%	Number	%
Until CO-Hb level normalized	28	58	7	15
For 24 h after CO exposure	5	10	1	2
For > 24 h after CO exposure	6	13	2	4
Until next morning	5	10	2	4
Until consciousness is regained	2	4	14	29
None set	0	0	22	46
Other	2	4	0	0

## Data Availability

The data used to support the findings of this study are available from the corresponding author upon request.
